# The effect of overcrowding in emergency departments on the admission rate according to the emergency triage level

**DOI:** 10.1371/journal.pone.0247042

**Published:** 2021-02-17

**Authors:** Hae Min Jung, Min Joung Kim, Ji Hoon Kim, Yoo Seok Park, Hyun Soo Chung, Sung Phil Chung, Ji Hwan Lee

**Affiliations:** Department of Emergency Medicine, Yonsei University College of Medicine, Seoul, Republic of Korea; Osakidetza Basque Health Service, SPAIN

## Abstract

Overcrowding in emergency departments is a serious public health issue. Recent studies have reported that overcrowding in emergency departments affects not only the quality of emergency care but also clinical decisions about admission. However, no studies have examined the characteristics of the patient groups whose admission rate is influenced by such overcrowding. This retrospective cohort study was conducted in a single emergency department between January 1 and December 31, 2018. Patients over 19 years old were enrolled and divided into three groups according to the degree of overcrowding—high, low, and non—based on the total number of patients in the emergency department. An emergency triage tool (the Korean Triage and Acuity Scale) was used, which categorizes patients into five different levels. We analyzed whether the degree of change in the admission rate according to the extent of overcrowding differed for each triage group. There were 73,776 patients in this study. In the analysis of all patient groups, the admission rate increased as the degree of overcrowding rose (the adjusted odds ratio for admission was 1.281 (1.225–1.339) in the high overcrowding group versus the non-overcrowding group). The analysis of the patients in each triage level showed an increase in the admission rate associated with the overcrowding, which was greater in the patient groups with a lower triage level (adjusted odds ratios for admission in the high overcrowding group versus non-overcrowding group: Korean Triage and Acuity Scale level 3 = 1.215 [1.120–1.317], level 4 = 1.294 [1.211–1.382], and level 5 = 1.954 [1.614–2.365]).

## Introduction

Overcrowding in the emergency department (ED) is a serious public health issue worldwide. Overcrowding in the ED is defined as a situation in which the demand for emergency services exceeds the ability of a department to provide quality care within acceptable time frames [1]. In 2018, the number of patients who were treated in EDs in Korea was 10,609,107, which was an increase of 1.76% compared to the previous year, and the number of patients admitted through EDs grew by 2.95% compared to the previous year. In contrast, the total number of ED beds in Korea was 6,945 in 2018, which decreased by 1.68% compared to the previous year [2]. These statistics indicate that the supply volume is not meeting the growing demand for emergency care, so the overcrowding in the EDs is an ongoing public health issue in Korea.

According to the national policy, the national medical insurance program covers almost all citizens and all medical institutions in Korea. Therefore, patients can freely choose the medical institution in which they wish to receive treatment [3]. This, coupled with the preferences of patients for large medical institutions, causes an overflow of patients in the EDs of those medical institutions.

This problem of overcrowding in the ED is caused by multiple factors, which include an increasing number of incoming ED patients (input factor), a shortage of ED resources (throughput factor), and the number of admitted patients who are waiting to move from the ED to a hospital ward (output factor) [4, 5]. Overcrowding in the ED directly decreases the quality of medical care, such as delaying medication time and increasing the mortality rate of admitted patients [6, 7]. It also causes various indirect problems, such as prolonged waiting times, decreased patient satisfaction, and profit loss by the medical institutions [4, 8, 9].

Ideally, medical decisions, such as triage decisions and admission decisions, should be based on the urgency of treatment and level of treatment needed by the patient. And these decisions should not be affected by the degree of overcrowding in the ED. The Emergency Severity Index and Canadian Triage and Acuity Scale, which are well-known triage tools, have warned clinicians that the “triage drift” phenomenon, which involves the adjustment of the triage level according to the degree of overcrowding in the ED, can have a negative effect on the patient’s prognosis [10–12]. However, previous studies have shown that medical decisions that are related to admission or the triage level may be distorted due to overcrowding in the ED [13, 14]. While previous research has investigated the relationship between overcrowding in the ED and the probability of admission, no studies have analyzed the characteristics of the admitted patients in the overcrowded ED environment.

An emergency triage tool is used to efficiently utilize limited medical resources in an overcrowded ED, and although various triage tools are used and differences exist in terms of their composition, a common factor is that the triage level is determined based on each patient’s overall medical condition, such as the urgency of the patient’s initial medical treatment and the severity of patient’s diseases [15, 16]. Therefore, this study aimed to investigate the effect of overcrowding in the ED on the admission rate, and especially how it differs depending on the triage level. Furthermore, this study was the first research done on study the problem of overcrowding in the ED in terms of both triage level and the admission rate.

## Materials and methods

### Study population and setting

This study was a retrospective cohort study that was conducted in one ED, which was visited by 100,000 patients in 2019. This hospital has 2,400 beds, and its ED is divided into an adult area and pediatric area. The adult practice area consists of 45 beds and 20 clinic chairs. This study included patients who were 19 years old or above and who visited the ED from January 1, 2018 to December 31, 2018.

In the research institution, the emergency physician provides primary care for all adult patients and makes decisions about admission and discharge. If the emergency physician determines that admission is necessary, the emergency physician decides the department in which the patient should be admitted and then consults with that department. And, final admission decision was made by physician who was requested consultation.

Some patients were excluded for the following reasons: canceled registration, visiting the ED other than for medical purposes, and omission of the analysis variables. In addition, patients who died before arrival or who expired in the ED were excluded because they were not considered for admission or discharge. The institutional review board of Severance Hospital approved this study, and informed consent was waived because this study was a retrospective study that analyzed previously collected medical records. (Approval number: 4-2020-0023)

### The Korean Triage and Acuity Scale

The Korean Triage and Acuity Scale (KTAS) was used as the emergency triage tool. The KTAS is a five-level triage tool that was developed based on the Canadian Triage and Acuity Scale [17]. The KTAS determines the patient’s priority for treatment by providing possible waiting time for treatment based on patient’s main symptoms, vital signs, and intensity of pain. In Korea, KTAS is used as a triage tool in all EDs and to triage, triage staff must complete six hours of a regular education session conducted by the Korean Society of Emergency Medicine according to the national policy. So all of the triage staff of this institution had completed regular education session. Ambulatory patients were triaged by nurses, and patients who were transferred by an ambulance were triaged by physicians.

### Study variables

The research data were extracted from the electronic medical record system of Severance Hospital in Korea, which data were completely anonymized during the extraction process. Among the study population, the date on which the last patient left the ED was January 6, 2019. Therefore, the period for collecting medical records was from January 1, 2018 to January 6, 2019. Basic demographic information was collected, such as age, sex, ambulance arrival, ED arrival time and medical/non-medical problems. If the patients’ problems were caused by external environmental factors, such as trauma and intoxication, this study defined these as non-medical problems. If the problems had not happened due to external factors, they were defined as medical problems. The complaint category variables were sorted by bodily system into 17 different categories based on the patients’ symptoms and they were used when the KTAS was employed to triage patients. When the data were analyzed, we included seven categories that were applied for more than 5% of the total number, and the remaining categories were labeled as “others.” The patient group who arrived during the day shift time (08:00–17:59), which is the regular bed assignment time, was defined as the day shift time arrival, and the patient group who arrived at other times was defined as the non-day shift time arrival. The research institute’s electronic medical recording system automatically records the number of patients in the adult area and pediatric areas every 10 minutes. This number includes the total number of patients in both the treatment and waiting areas; hence, these data were used. The number of patients at the time (the minute of each patient’s arrival time was rounded-up from the original time) was used as the standard value in this study. The patients were divided into three different groups based on the tertiles of the number of patients: a high overcrowding group (HOG), low overcrowding group (LOG), and non-overcrowding group (NOG). The outcome variable was whether the patient was admitted or not. Admission was defined as the situation wherein a patient was actually admitted to an inpatient bed. Therefore, even if the patient was in the ED for a long time, and if the destination at the time of leaving the ED was an inpatient bed, it was classified as admission.

### Statistical analysis

SAS (version 9.3, SAS Institute Inc., Cary, NC, USA) was used for the statistical analysis. Categorical variables were presented as *n* (%), and continuous variables were presented as the median (inter-quartile range). To compare the demographic data, the chi-square test was used for the categorical variables. The Kruskal-Wallis test was used for the continuous variables because they did not have a normal distribution. To analyze the correlation between ED overcrowding and the admission rate according to the KTAS level, the entire patient group was divided into five groups according to the KTAS level, and an individual analysis was performed. By using logistic regression, the results were presented as adjusted odds ratios, which were obtained after applying the adjustment variables, such as age, sex, medical/non-medical problem, use of an ambulance, complaint category, and day shift time arrival / non-day shift time arrival. When comparing the degree of overcrowding, the NOG was set as the reference group. In addition, the linear predictor of each KTAS group was extracted by using logistic regression not only for the group comparisons but also to calculate the changes in the admission rate according to the increasing number of patients in the ED.

## Results

**Fig 1:**
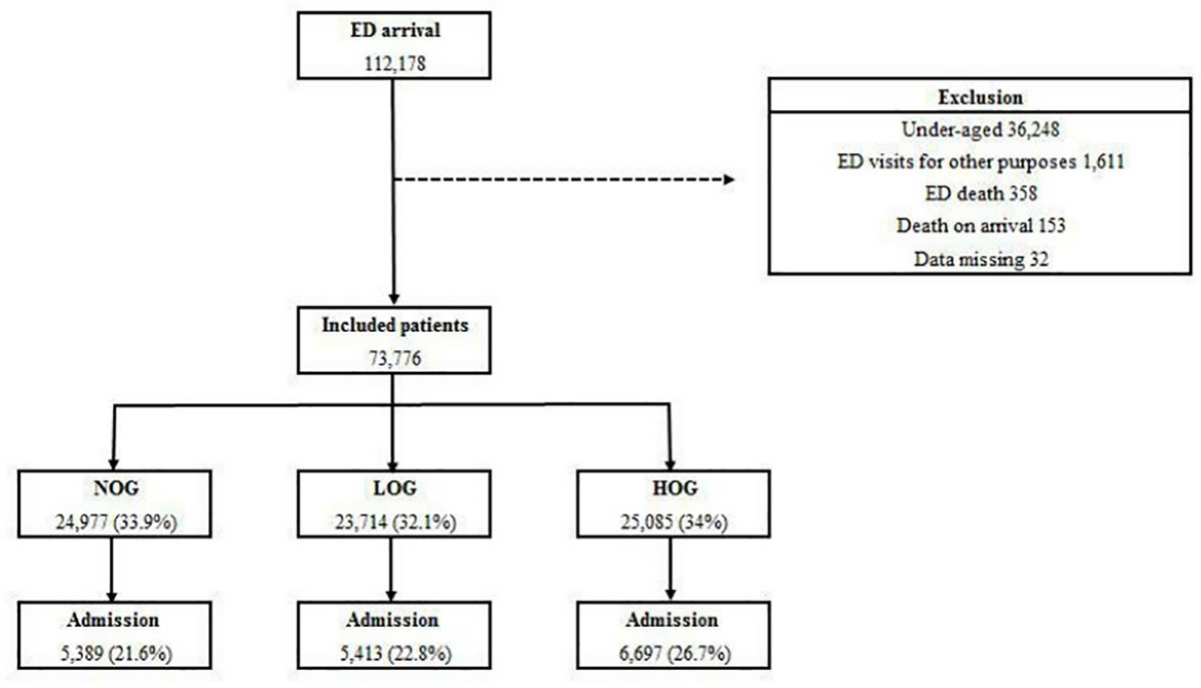
Number of included patients. ED, emergency department; NOG, non-overcrowding group; LOG, low overcrowding group; HOG, high overcrowding group.

During the study period, the total number of patients who visited the ED was 112,178. Of these, 36,248 patients who were aged under 19 years, 1,611 patients who visited the ED other than for medical purposes, and another 543 patients who met the aforementioned exclusion criteria, were excluded from this study (Fig 1). The final number of enrolled patients was 73,776. The number of admitted patients was 17,499 (23.7%). The number of patients in the ED at any given time ranged from 12 to 98. Based on the number of patients in the ED at the time of arrival of each patient, patients were classified into three different groups. If the number of patients ranged from 12 to 49, they were classified into NOG. If it ranged from 50 to 62, they were classified into LOG, and if it ranged from 63 to 98, they were classified into the HOG. The number of patients in each group was 24,977 (33.9%), 23,714 (32.1%), and 25,085 (34%), respectively. The collected information is presented in Fig 1.

### Characteristics of the patients

The median age of the patients was 54 years (24–69), and there were 39,655 female patients (53.8%). When comparing the groups, patients in the HOG had the highest median age and this group had the lowest proportion of patients who had non-medical problems. In addition, the admission rate was high. The proportion of patients classified into KTAS levels 1–3, which are considered to be relatively high-emergency grades, was 33.5% in the HOG, 30.3% in the LOG, and 30.9% in the NOG. The demographic information is provided in Table 1.

**Table 1:** Demographic information of each overcrowding group.

	Total	NOG	LOG	HOG	P-value
	N = 73,776	N = 24,977	N = 23,714	N = 25,085	
Age (years)	54 (24,69)	53 (33,68)	53 (34,68)	55 (36.70)	<0.001
Total number of ED patients, median (n)	56 (44,66)	41 (35,46)	56 (53,59)	71 (66,76)	<0.001
Female, n (%)	39,655 (53.8)	13,367 (53.5)	12,868 (54.3)	13,420 (53.5)	0.157
KTAS level, n (%)					<0.001
1	838 (1.1)	280 (1.1)	258 (1.1)	300 (1.2)	
2	5,711 (7.7)	1,921 (7.7)	1,747 (7.4)	2,043 (8.1)	
3	16,770 (22.7)	5,516 (22.1)	5,190 (21.9)	6,064 (24.2)	
4	40,280 (54.6)	13,815 (55.3)	13,118 (55.3)	13,347 (53.2)	
5	10,177 (13.8)	3,445 (13.8)	3,401 (14.3)	3,331 (13.3)	
KTAS category					<0.001
Gastrointestinal	14,885 (20.2)	5,347 (21.4)	4,701 (19.8)	4,837 (19.3)	
General*	12,107 (16.4)	3,751 (15)	3,911 (16.5)	4,445 (17.7)	
Neurology	10,814 (14.7)	3,685 (14.8)	3,416 (14.4)	3,713 (14.8)	
Cardiovascular	7,151 (9.7)	2,253 (9)	2,251 (9.5)	2,647 (10.6)	
Orthopedics	7,000 (9.5)	2,280 (9.1)	2,283 (9.6)	2,437 (9.7)	
Respiratory	5,347 (7.2)	1,660 (6.6)	1,636 (6.9)	2,051 (8.2)	
Skin	5,103 (6.9)	1,847 (7.4)	1,722 (7.3)	1,534 (6.1)	
Others**	11,369 (15.4)	4,154 (16.6)	3,794 (16)	3,421 (13.6)	
Non-medical problem	13,096 (17.8)	4,534 (18.2)	4,407 (18.6)	4,155 (16.6)	<0.001
Ambulance arrival, n (%)	18,281 (24.8)	6,893 (27.6)	5,747 (24.2)	5,641 (22.5)	0.298
Non-day shift time arrival, n(%)	36,315 (49.2)	13,116 (52.7)	12,268 (51.7)	10,881 (43.4)	<0.001
Admission, n (%)	17,499 (23.7)	5,389 (21.6)	5,413 (22.8)	6,697 (26.7)	<0.001

NOG, non-overcrowding group; LOG, low overcrowding group; HOG, high overcrowding group; ED, emergency department; KTAS, Korean triage and acuity scale

*, not limited to a specific system

**, other categories not listed.

### Differences in the admission rates of each ED overcrowding group

The analysis of all of the patients indicated that the admission rate increased as the ED overcrowding grew. The odds ratios for admission gradually rose to 1.106 (95% confidence interval [CI]: 1.057, 1.159) and 1.281 (1.225, 1.339), respectively, as the overcrowding increased to the LOG and HOG compared to the NOG (*p* < 0.001). In the analysis according to the KTAS level, the odds ratio for admission in KTAS group 2 tended to rise as the overcrowding increased, although there was no statistically significant difference. However, in the groups with a KTAS level of 3 or lower, the admission rate increased as the overcrowding rose. As the ED overcrowding changed from non to low and high, the odds ratios for admission were 1.109 (1.020, 1.206) and 1.215 (1.120, 1.317) in KTAS group 3, 1.100 (1.027, 1.178) and 1.294 (1.211, 1.382) in KTAS group 4, and 1.407 (1.149, 1.722) and 1.954 (1.614, 2.365) in KTAS group 5, respectively, which were statistically significant. Table 2 presents the odds ratios for admission according to the overcrowding groups.

**Table 2:** Logistic regression between the degree of overcrowding and admission rate in each group of the Korean Triage and Acuity Scale.

	All		KTAS 1		KTAS 2		KTAS 3		KTAS 4		KTAS 5	
	OR (95% CI)	p-value	OR (95% CI)	p-value	OR (95% CI)	p-value	OR (95% CI)	p-value	OR (95%CI)	p-value	OR (95%CI)	p-value
Overcrowding												
Low	1.106 (1.057–1.159)	<0.001	0.799 (0.540–1.182)	0.261	1.097 (0.953–1.263)	0.196	1.109 (1.020–1.206)	0.016	1.100 (1.027–1.178)	<0.001	1.407 (1.149–1.722)	0.001
High	1.281 (1.225–1.339)	<0.001	0.956 (0.649–1.408)	0.821	1.070 (0.934–1.226)	0.331	1.215 (1.120–1.317)	<0.001	1.294 (1.211–1.382)	<0.001	1.954 (1.614–2.365)	<0.001
Age (years)	1.021 (1.019–1.022)	<0.001	1.006 (0.997–1.016)	0.203	1.014 (1.011–1.017)	<0.001	1.013 (1.011–1.015)	<0.001	1.022 (1.020–1.023)	<0.001	1.022 (1.018–1.027)	<0.001
Female	0.661 (0.638–0.686)	<0.001	0.844 (0.614–1.161)	0.298	0.702 (0.627–0.787)	<0.001	0.685 (0.641–0.732)	<0.001	0.717 (0.679–0.758)	<0.001	0.609 (0.523–0.710)	<0.001
Non-medical problem	0.498 (0.464–0.534)	<0.001	0.599 (0.289–1.243)	0.169	1.149 (0.889–1.484)	0.290	0.843 (0.718–0.989)	0.036	0.491 (0.444–0.543)	<0.001	0.403 (0.323–0.502)	<0.001
Ambulance arrival	2.277 (2.186–2.372)	<0.001	2.447 (1.699–3.525)	<0.001	1.768 (1.563–2.000)	<0.001	1.630 (1.520–1.747)	<0.001	1.494 (1.395–1.601)	<0.001	1.393 (1.116–1.738)	0.003
Complaint category												
Gastrointestinal	2.996 (2.792–3.214)	<0.001	2.333 (0.886–6.287)	0.094	6.785 (4.650–9.899)	<0.001	3.073 (2.667–3.541)	<0.001	3.159 (2.867–3.479)	<0.001	3.442 (2.395–4.947)	<0.001
General	2.402 (2.233–2.584)	<0.001	0.959 (0.376–2.445)	0.930	2.921 (2.261–3.773)	<0.001	2.541 (2.187–2.953)	<0.001	2.591 (2.335–2.875)	<0.001	3.744 (2.807–4.994)	<0.001
Neurology	1.502 (1.392–1.621)	<0.001	0.235 (0.103–0.532)	0.001	1.690 (1.329–2.148)	<0.001	0.752 (0.658–0.859)	<0.001	1.191 (1.036–1.369)	0.014	1.739 (0.753–4.015)	0.195
Cardiovascular	1.678 (1.546–1.821)	<0.001	0.501 (0.217–1.157)	0.106	1.346 (1.066–1.699)	0.012	0.862 (0.729–1.018)	0.081	1.471 (1.292–1.674)	<0.001	5.161 (3.434–7.757)	<0.001
Orthopedics	1.518 (1.386–1.663)	<0.001	0.387 (0.043–3.445)	0.394	2.463 (1.519–3.994)	<0.001	1.266 (1.001–1.602)	0.049	2.260 (1.999–2.556)	<0.001	3.495 (2.574–4.747)	<0.001
Respiratory	3.226 (2.967–3.508)	<0.001	0.449 (0.189–1.068)	0.070	5.634 (4.179–7.597)	<0.001	2.444 (2.108–2.834)	<0.001	2.169 (1.882–2.499)	<0.001	1.179 (0.701–1.983)	0.535
Skin	0.385 (0.327–0.454)	<0.001	0*	0*	0.191 (0.087–0.417)	<0.001	0.646 (0.430–0.970)	0.035	0.618 (0.493–0.775)	<0.001	0.667 (0.444–1.003)	0.052
Non-day shift time arrival	0.672 (0.647–0.697)	<0.001	1.283 (0.925–1.780)	0.136	0.808 (0.720–0.906)	<0.001	0.742 (0.692–0.795)	<0.001	0.628 (0.594–0.665)	<0.001	0.466 (0.393–0.551)	<0.001

KTAS, Korean Triage and Acuity scale; OR, odds ratio; CI, confidence interval

The reference category for the degree of overcrowding is “non-overcrowding group”

The reference category for the complaint category is “Others.”

*, There were no admitted patients among patients triaged as KTAS 1 through the skin category; therefore the statistical value was not calculated.

### Differences in the admission rates according to the increase in the number of patients in the ED

**Fig 2:**
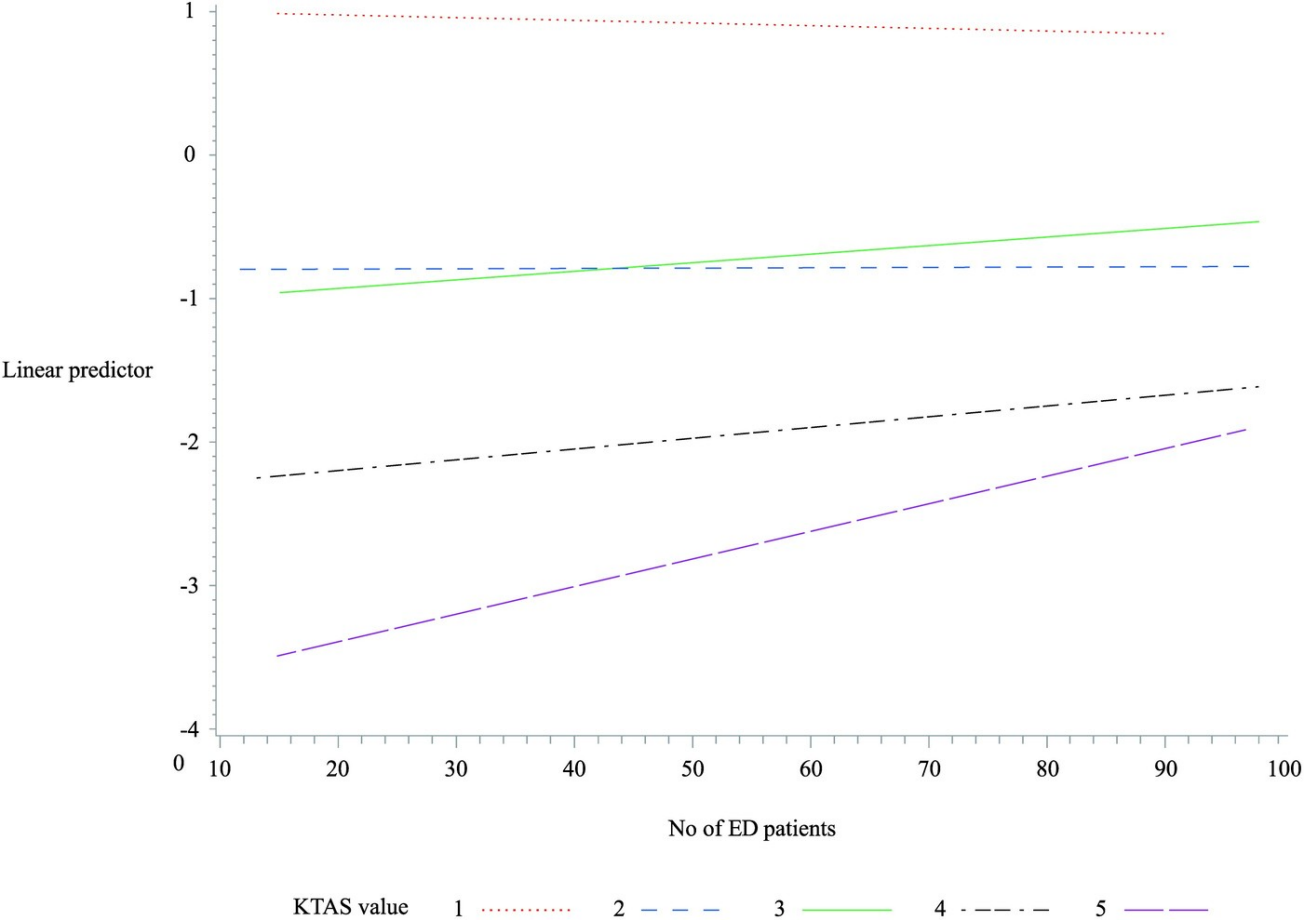
Linear predictor for admission using the Korean Triage and Acuity Scale value. KTAS, Korean Triage and Acuity Scale; ED, emergency department.

The difference in the admission rates due to the increase in the number of existing patients in the ED was the same as in the above inter-group comparison. No statistically significant correlations were found in KTAS groups 1 and 2 for changes in the admission rate as the number of existing patients grew; however, a statistically significant relationship was found in KTAS groups 3 to 5 (*p* < 0.001). In addition, the lower the KTAS level, the greater the increase in the admission rate as the number of existing ED patients rose (the coefficient of KTAS level 3 = 0.00576, the coefficient of KTAS level 4 = 0.00747, and the coefficient of KTAS level 5 = 0.01930) (Fig 2).

## Discussion

This study found that the greater the overcrowding in the ED, the greater the likelihood of the patients being admitted. In addition, the findings indicated that the impact of the overcrowding on the admission rate was greater in the patient groups with a lower triage level (KTAS 3, 4, and 5).

Previous studies have analyzed the correlation between overcrowding in the ED and the admission rates. In 2019, Chen et al. reported that the odds ratio of a patient being admitted when the number of patients in the ED increased by one was 1.007 (95% CI: 1.006, 1.008) [13]. In addition, in 2017, Gorski et al. stated that as the number of patients waiting for ED treatment grew, the odds ratio to be admitted was 1.011 (95% CI: 1.001, 1.020) [14]. In our study, as the total number of ED patients increased, the odds ratio of the patients being admitted was 1.007 (95% CI: 1.006, 1.008), which is similar to that of previous studies. Therefore, this result strongly supports the previous research.

Some studies have explained that the cause of this phenomenon is the admission of patients in the “gray zone,” which is in the boundary between admission and discharge, when the ED is overcrowded. The changes in the physicians’ decision-making process can be explained as follows. As the number of ED patients rises, the amount of patients that the physicians have to deal with simultaneously increases, which causes information overload for the doctors [14]. Along with this, their mental resources become depleted when repeated decisions related to the ED patients’ dispositions are made without adequate resting time [18]. Both of these situations lead to the decision-makers avoiding difficult choices and simplifying their decision-making. As a result, physicians are more likely to avoid additional assessments, judgments, or discharge plans that need to be implemented for safe discharge and they are more likely to make easier admission decisions.

Although previous studies have reasonably suggested the above mentioned reasons, they have not been able to provide information about the actual changes in the admission rate of patients with certain characteristics. Emergency triage tools evaluate the patients’ overall medical condition, such as the urgency and severity of their problem; however, the emergency triage level is not an absolute measure of the need for admission. Nevertheless, in general, when emergency physicians use the KTAS, which is a five-step emergency classification triage tool, they recognize that KTAS levels 1 and 2 are severe emergency patients, KTAS levels 3 and 4 are general emergency patients, and those with KTAS level 5 are non-emergency or mild illness patients. Therefore, from the perspective of emergency medical personnel, and assuming that patients considered to be in the gray zone are included in KTAS groups 3 and 4, and this study was conducted to confirm the hypothesis that was suggested from the previous studies. However, our results confirmed that the admission rate of non-emergency patients who were triaged as level 5, who were not assumed to be gray-zone patients, also increased. This can be due to the influence of the characteristics of the patient group in the hospital where this research was conducted. This study was performed in a tertiary referral hospital where the ratio of patients who have severe underlying diseases, such as cancer, autoimmune diseases, and organ transplants, is comparatively very high. However, in the KTAS triage tool used in this study, patients’ underlying conditions are not considered, other than immunosuppressed conditions in fever patients and hemorrhagic tendency with bleeding [17]. Therefore, the degree of the emergency of the patients visiting the ED with severe underlying chronic symptoms or diseases could be relatively under-triaged. In fact, among the 818 patients who were triaged as KTAS level 5 and admitted to the hospital, about 270 had a history of malignant tumor, and about 40 were identified as having received transplantations. Therefore, it is assumed that there were a number of gray-zone patients among those who were classified into the KTAS group 5.

There are several limitations to this study. First, this research was conducted with a sample from a single institution; therefore, the results of this study cannot be generalized to all medical institutions. Therefore, additional research is needed that involves multi-center studies. Second, the degree of ED overcrowding is usually evaluated through various factors, such as the number of ED beds and hospital beds, the number of patients in the ED, the number of admitted patients, the number of respirators in the ED, the longest admitted time, and the waiting room time of the last patient [19]. However, in this study, overcrowding was assessed only by the number of ED patients, and other factors were not evaluated. Third, the physicians who made the admission decisions changed during the study period according to the regular ED duty schedule of the research institution. Therefore, we cannot rule out the possibility that differences in the individual physicians’ inclinations about the patients’ admission influenced their decision-making. Finally, in this study, only the trend in the patients’ admission rate was confirmed, and the appropriateness of each individual admission was not evaluated.

## Conclusions

This study found that there is a positive relationship between overcrowding in the ED and the admission rate of patients, and this effect increases for those patients who are triaged as having a lower acuity level. These results suggest that overcrowding in the ED possibly causes multiple problems, such as the unnecessary consumption of medical resources and needless admissions.

## Supporting information

S1 Data Raw data used in this study. (XLSX)
